# (Dis)Integrated Care? Lessons from East London

**DOI:** 10.5334/ijic.5432

**Published:** 2020-10-19

**Authors:** Sonia Bussu, Martin Marshall

**Affiliations:** 1 Manchester Metropolitan University, UK; 2 UCL, UK

**Keywords:** integrated care, working routines, community care, organisational change, vertical and horizontal integration

## Abstract

**Introduction:**

This paper examines one of the NHS England Pioneers programmes of Integrated Care, which was implemented in three localities in East London, covering the area served by one of the largest hospital groups in the UK and bringing together commissioners, providers and local authorities. The partners agreed to build a model of integrated care that focused on the whole person. This qualitative and participatory evaluation looked at how an ambitious vision translated into the delivery of integrated care on the ground. The study explored the micro-mechanisms of integrated care relationships based on the experience of health and social care professionals working in acute and community care settings.

**Methods:**

We employed a participatory approach, the Researcher in Residence model, whereby the researcher was embedded in the organisations she evaluated and worked alongside managers and clinicians to build collaboration across the full range of stakeholders, develop shared learning, and find common ground through competing interests, while trying to address power imbalances. A number of complementary qualitative methods of data generation were used, including documentary analysis, participant observations, semi-structured interviews, and coproduction workshops with frontline health and social care professionals to interpret the data and develop recommendations.

**Results:**

Our fieldwork exposed persistent organisational fragmentation, despite the dominant rhetoric of integration and efforts to build a shared vision at senior governance levels. The evaluation identified several important themes, including: a growing barrier between acute and community services; a persisting difficulty experienced by health and social care staff in working together because of professional and cultural differences, as well as conflicting organisational priorities and guidelines; and a lack of capacity and support to deliver a genuine multidisciplinary approach in practice, despite the ethos of multiagency being embraced widely.

**Discussion:**

By focusing on professionals’ working routines, we detailed how and why action taken by organisational leaders failed to have tangible impact. The inability to align organisational priorities and guidelines on the ground, as well as a failure to acknowledge the impact of structural incentives for organisations to compete at the expense of cooperation, in a context of limited financial and human resources, acted as barriers to more coordinated working. Within an environment of continuous reconfigurations, staff were often confused about the functions of new services and did not feel they had influence on change processes. Investing in a genuine bottom-up approach could ensure that the range of activities needed to generate system-wide cultural transformation reflect the capacity of the organisations and systems and address genuine local needs.

**Limitations:**

The authors acknowledge several limitations of this study, including the focus on one geographical area, East London, and the timing of the evaluation, with several new interventions and programmes introduced more or less simultaneously. Some of the intermediate care services under evaluation were still at pilot stage and some teams were undergoing new reconfigurations, reflecting the fast-pace of change of the past decade. This created confusion at times, for instance when discussing specific roles and activities with participants. We tried to address some of these challenges by organising several workshops with different teams to co-interpret and discuss the findings.

## Introduction

Health systems across advanced economies face increasing challenges, as they try to improve population health and enhance patient experience, while ensuring cost-effectiveness, often in a context of financial austerity. There is a growing consensus that integrated care can help address some of these challenges; however, the evidence base to date is mixed [1,2] and the claims of policymakers in favour of integrated care are not always confirmed by large-scale evaluations or recent systematic reviews [3,4,5,6]. Randomised stepped-wedge trials in primary care on the introduction of predictive risk stratification models found that the introduction of the PRISM (Profiling Risk, Integrated Care, Self-Management) model of care increased emergency episodes, hospitalisation and costs without clear evidence of benefits to patients [7]. Evidence that pooled budgets between health and social care organisations or the use of multidisciplinary teams and joint commissioning of services have significant impact on emergency hospital admissions or cost savings is also weak [8]. While the principles of better coordination and holistic care are hard to argue against, disappointing outcomes might result from the gap between the high-level vision of integrated care and the reality on the ground, with growing demand for complex care but limited capacity and profound organisational and professional differences that hinder efforts at integration. A growing theme in the integrated care literature concerns the role of relationship building to increase trust between different roles, who may have divergent understandings of what integrating care entails, and efforts at bridging across professional and cultural divides, such as those existing between health and social care [9,10,11]. However, this literature also appears to show limited evidence that inter-professional relationships and communication are enhanced through integrated care. As an example, delayed discharges from hospital appear to depend not so much on professional relationships between staff working in acute or community care settings, but rather on organisational factors [12].

The focus of much of this literature has often been either on cost-benefit analysis or different models of integrated care interventions and the aspects that should guide service design to facilitate care integration [13]. Less work has been devoted to identifying the micro-mechanisms that might encourage or hinder the delivery of systemic integration. These refer to a range of dynamics at the micro-level, from tensions between new integrated care services and existing teams to the impact of care integration on different working routines and professional cultures, as well as capacity, resources and expectations across organisations at the point of delivery [13]. A better understanding of these micro-mechanisms might help shed some light on the persisting gap between the rhetoric of integrated care, or integrated care as imagined by policymakers and senior management, and the reality of care as delivered by frontline clinicians and other health and social care professionals [14].

In England, there has been significant investment in integrated care initiatives, particularly at the local level, from NHS foundation trusts and local authorities [15,16,17]. This paper focuses on one of 14 successful applicants across England to achieve pioneer status for integrated care in May 2013 [18]. The areas designated by the NHS in England (NHSE) as pioneers were expected to develop innovative ways to address local barriers to integrated care delivery and help identify national-level barriers. The Pioneers had access to support from a range of national and international experts but received limited additional funding from NHSE (£20,000 initially, later supplemented with a further £90,000) [19]. This qualitative and participatory evaluation covered the three East London municipalities that came together to form an integrated care programme and achieved pioneer status. The purpose of the evaluation was to understand how the high-level principles and visions translated into the delivery of integrated care, through a focus on micro-mechanisms and working routines.

The literature has often identified two persisting gaps in integrated care delivery: poor understanding of clinical collaboration across levels of healthcare delivery and limited attention to the individual experience of different forms of professional work [10,20]. Thus, a focus on organisational routines can help understand these relationships and evidence patterns of resistance to, and sustainability of, change. Routines are defined as recurrent, collective, and interactive behaviour patterns, which are specific to a particular local context and sets of relations and help coordinate work [1,21]. Routines are often path-dependent and feedback effects such as competency traps can contribute to understanding organisation change, as they show how actors tend to make decisions based on prior experience [22].

This paper provides an in-depth analysis of the perceptions of health and social care professionals working within acute and community settings in the three East London municipalities, and their experience of integrated care. The aim is to evidence how the new integrated care services interacted, and conflicted, with existing working routines. Within an environment of ongoing reforms, these professionals were asked to alter their routines; however, they felt design of the new services often ignored existing routines or lacked thorough consideration of potential unintended consequences. The next section describes our methodological approach, followed by a description of the case, presentation of the main results, and a discussion of the findings and implications for policy and practice.

## Methods

### Research design

A qualitative evaluation of the East London pioneer programme had already been carried out between September 2014 and August 2016 [23] and looked at different ways of understanding – and motivations for – integrated care across the organisations involved. That initial work highlighted how, although governance structures had been set up, a deep chasm remained between strategic thinking and operational delivery. This study built on those findings, by evaluating how the new integrated services were embedding within and across acute and community care.

The paper presents findings on two pathways, admission avoidance and hospital discharges. These were identified as priorities by the organisations involved in the study, which helped coproduce the research protocol; the new integrated services were involved in one or both pathways. The evaluation examined three new integrated services in each locality: Rapid Response (RR), Discharge to Assess (D2A), and the new community teams. RR and D2A aimed to improve coordination between the hospital and community services, or what has been defined in the literature as vertical integration. The new integrated community teams, called Extended Primary Care teams (EPCTs) in Locality A and B and Integrated Care Teams (ICTs) in Locality C, were envisaged to strengthen integration across different healthcare roles in the community and between health and social care, or horizontal integration [24,25].

The evaluation employed a participatory approach to research, the Researcher-in-Residence model [26,27,28], where the researcher is embedded in the organisations under study. The rationale was to build collaboration across the full range of stakeholders, with a focus on solving practical problems; initiating change through reflection, greater understanding and shared learning; and finding common ground through competing interests, while trying to address power imbalances. The aim was for the researcher and the participants to ‘co-create’ knowledge on how to achieve better coordination of care. The Researcher-in-Residence model offered opportunities to build research capacity within the participating organisations. In line with the participatory ethos of the model, frontline health and social care professionals participated in coproduction workshops to co-interpret emergent findings; share feedback on whether the findings resonated with their experience; provide clarifications and updates on recent developments; and co-produce suggestions to address the challenges identified. We reflect in more detail on the Researcher-in-Residence model and the new ethical issues it raises elsewhere [29].

### Data collection

The project was approved by the UCL research ethics in August 2017 and gained NHS Health Research Approval in October 2017, following a long scoping phase (May–September 2017). A number of complementary qualitative methods of data generation were used including documentary analysis, participant observations (over 200 hours), 81 semi-structured interviews with frontline health and social care professionals (October 2017-February 2018), and coproduction workshops (April–June 2018). Interviews aimed to elicit insights on how new integrated services worked, whether participants were clear about their different functions, and what impact these new services had on different roles and patterns of collaboration across the organisations involved. Interviewees were identified using a broadly purposive strategy, ensuring a balanced mix of different roles from the new integrated care services, as well as other roles from acute, community, and social care involved in the two pathways under study (e.g. nurses; occupational therapists and physiotherapists; medical staff; care navigators; service leads/team managers) [30]. The sample also included actors from the voluntary sector working with the new integrated services (e.g. AgeUK). Interviews lasted between 45 minutes and one hour and 30 minutes and were carried out at the interviewee’s place of work. A table in Appendix details the teams involved in the study and participants’ roles.

### Data analysis

Deductive-inductive thematic analysis was adopted as a strategy for data analysis to develop an understanding of how pathways of admission avoidance and discharge from hospital happen on the ground and how the new integrated services function and collaborate. The research team developed codes on participants’ perceptions of existing barriers and enablers to working with colleagues from different organisations and/or in different parts of the care system. The focus of the analysis was on existing working routines to explore how these have changed and/or developed following recent integrated care initiatives and what participants felt were the drivers of, and hinders to, change. Codes helped identify patterns within the data, while thematic maps aided the generation of themes. Some of the main themes that emerged, as examined in the following sections, reflected anxieties about “too many new services and confusion on their functions”; “the pressure on understaffed teams to provide holistic care without adequate resources”; “conflicting priorities of different organisations which affected staff at the point of the delivery”; and “the difficulty to build relationships of trusts in a context of high turnover”.

Provisional explanatory accounts and maps of the pathways in each locality (see pathway maps in Appendix) were discussed and interpreted with participants through a series of coproduction workshops, as described above. Analysis was therefore a recursive process, with movement back and forth between different phases to refine collective interpretation and understanding of the data.

## Description of the case: new integrated teams within admission avoidance and discharge pathways

All three localities are characterised by significant levels of deprivation, in a context of growing and ethnically diverse populations and increasing demand for complex care – e.g. long-term conditions, such as diabetes. Together they cover the area served by one of the largest hospital groups in the UK. The integrated care programme brought together Clinical Commissioning Groups (CCGs), providers (NHS Trusts) and local authorities of the three municipalities. Table 1 lists the programme’s partner organisations.

**Table 1:** Programme partners.

Locality A	Locality B	Locality C

Clinical Commissioning group	Clinical Commissioning group	Clinical Commissioning group
Local Authority	Local Authority	Local Authority
Acute Trust (mainly covering acute services across all three localities)		
Community services Trust for Localities A and B		Community services Trust for Locality C

**Figure 1:**
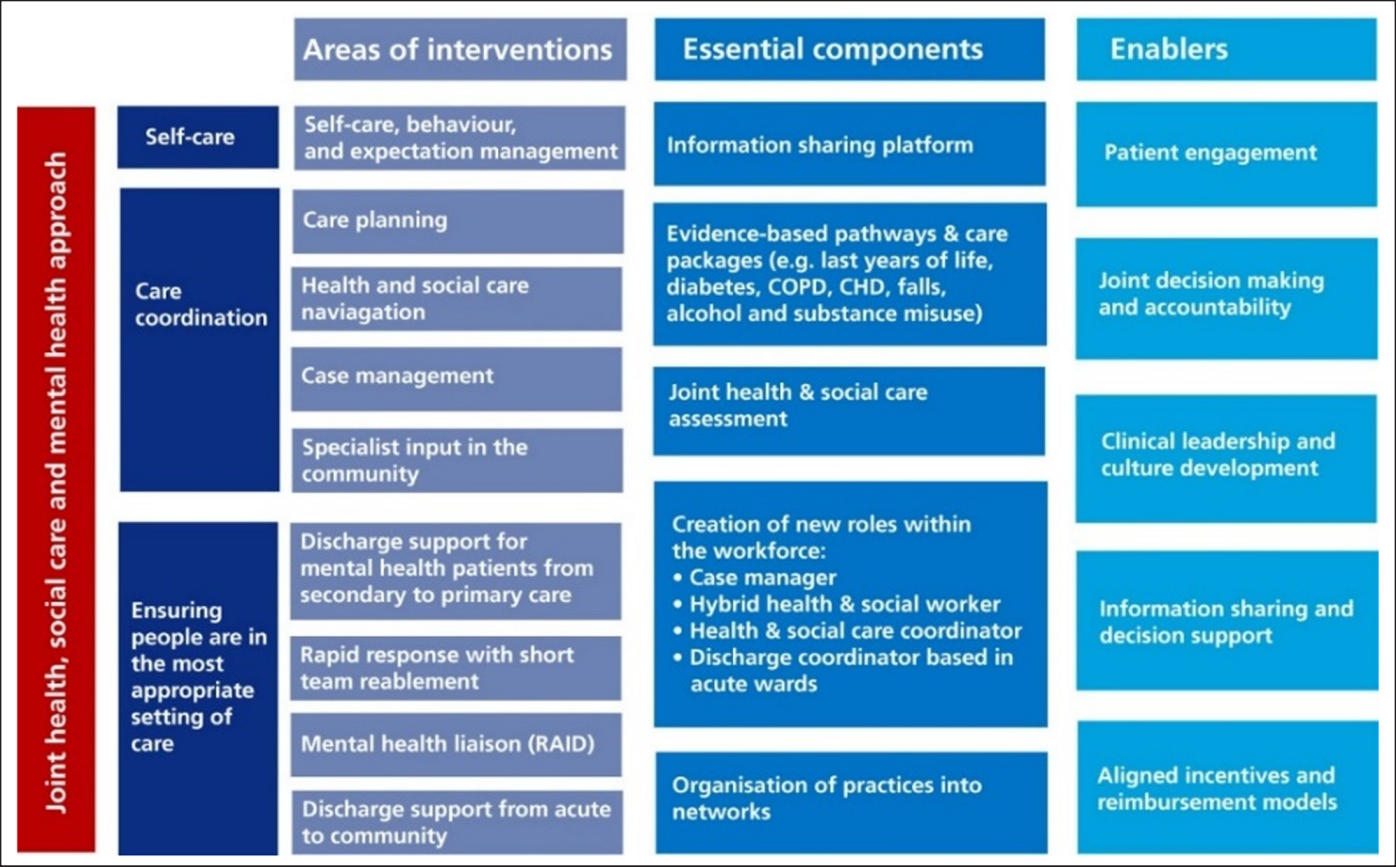
Areas of interventions for Pioneer Integrated Care Programme in East London.

The partners agreed to build a model of integrated care that looked at the whole person, i.e. physical and mental health and social care needs. They agreed a common set of principles and areas of intervention, which informed their approach to integrated care (Figure 1). The overarching aim was to reduce non-elective admissions, through developing risk-stratification tools to identify high-risk patients and by introducing intermediate care services, which could respond to urgent calls in the patient’s home, such as Rapid Response (RR) and Discharge to Assess (D2A), and integrated community teams to provide holistic care to patients in the community.

Compared to the other two municipalities, Locality A already had a long history of collaboration at governance level between the health and social care systems. It is comprised of four areas with a population of 60000–80000 each. Each area includes a multi-professional community care team (EPCT) providing community nursing and therapies for patients aged over 18 and resident in the borough. Locality B has a similar model to Locality A with EPCTs covering four areas, with a population of approximately 80,000 each, and incorporating eight General Practitioner (GP) clusters. In Locality C, with an overall population of 276,000, the community service provider carried out a restructuring of community health services based on the distinction between planned and unplanned care. The three multiagency Integrated Care Teams (ICTs) would focus on planned care and provide adult community health services. These community teams across all three municipalities were expected to improve coordination between nurses, therapists and social workers and were envisaged to play a crucial role in reducing hospital admissions. Both EPCTs and ICTs comprise of district nurses (DN), occupational therapists (OT) and Physiotherapists; Locality C’s ICTs also include community matrons. The EPCTs in Localities A and B introduced the role of care navigators. These are non-clinicians who support complex adults and help them navigate the health and social care system, for instance by ensuring they receive adequate support to attend hospital appointments and have access to the benefits and care they are entitled to. At piloting stage, each community team had a dedicated social worker co-located with healthcare professionals; in most cases, limited capacity of social services meant this close relationship was difficult to sustain in the long-term, with social workers eventually only attending monthly multi-disciplinary meetings (MDTs) at the local general practice (GP).

In each locality, a RR team was also established as part of the admission avoidance strategy. RR is a nurse-led service that delivers unplanned and urgent care services in the patient’s home to avoid non-elective hospital admissions. It provides a rapid assessment and immediate treatment and represents an alternative to hospital admission when acute episodes of care are required that can be managed within the community, in a clinically appropriate way. Generally based within the hospital but managed by community services Trusts, RR teams aim to contribute to bridging the gap between acute and community care. However, despite high face validity, the effectiveness of RR remains unclear [31,32]. Whereas the RR service was nurse-led in all three localities, in Locality A and C it included healthcare assistants and worked closely with physiotherapists and OTs from other services; in Locality B a physiotherapist, an OT, a GP, and four geriatricians from the local hospital were also part-time members of the team.

In order to strengthen the discharge pathway, all three localities introduced a D2A service, with the aim to facilitate faster discharge of medically fit patients and provide therapy and social care assessment in the patient’s home. This service generally includes physiotherapists and OTs working closely with social services’ reablement teams (delivering restorative care and helping people with poor physical and mental health to re-learn skills for daily living) and care providers to offer ongoing support (up to 6 weeks) in the patient’s home and increase levels of function and independence.

## Results

Fieldwork revealed a high degree of organisational fragmentation, which inevitably affected collaboration and coordination at the point of delivery, at times increasing risks of overlap and duplication. The rest of this section presents findings relating to each of the pathways evaluated.

### Admission avoidance pathway

The new RR teams were perceived to be playing a positive role across all three localities, but participants in the study often saw them as “picking up the pieces” from other parts of the system. Albeit making a substantive difference to patients’ care in the short-term, the new service appeared to act as remedial support within a dysfunctional system. As an example, the RR team’s flexible inclusion criteria generated confusion about the boundaries of the service and raised expectations from district nurses (DN) in community teams that RR would regularly respond to patients that would normally be on a community team’s caseload (e.g. wound dressings; unscheduled visits). This affected the service’s capacity to respond to those emergencies, which it was initially designed to address, as illustrated by the interview excerpt below:

We’d come to about four o’clock… three or four o’ clock and then one nurse finishes at five, and then the last nurse is left to work until eight. So they’ve got their list and we had about fourteen calls that day. So a lot of them were catheter-related calls […] that traditionally would have gone to district nurses. So they’re all going out doing their visits plus the calls and then we had one call from a GP […] saying that they’ve got this patient who has got dementia, behaviour’s getting worse, probably has a UTI but she has a catheter and she’s always pulling the catheter out. […] So, it’s a call that we could have gone with our bladder scanner, scanned the retention, you know, done a full assessment. […] But we couldn’t deal with it because the last nurse that was working was already going to do a simple blocked catheter (RR nurse)

Several participants felt a holistic approach to community care was necessary to respond to complex care demand, and this relied on a strong relationship between DNs, therapists, GPs, and social workers. These relationships experienced a number of familiar challenges, including limited resources; understaffed healthcare teams; broken communication between community teams, GPs, and social workers, whereby staff regularly struggled to get hold of other professionals; pressure on staff from increasing administrative tasks with less time for direct contact with patients. High turnover and widespread use of agency staff also hindered relationship building. Whereas senior management’s rhetoric often emphasised the need to reduce hospital admissions, and therefore costs for acute Trusts, and shift care to the community, the evaluation highlighted how community care continues to lack the financial and human resources that would enable it to step in effectively.

There can be very unrealistic referrals [from the hospital]. So three times a day dressing changes. Things like that that could be facilitated in a ward, but not facilitated here (DN).

The multidisciplinary ethos of integrated community teams was endorsed by both health and social care professionals; however, many felt they still worked in silos and opportunities to coordinate joint assessments and visits were rare, due to heavy caseloads. Furthermore, different organisational priorities and pressures on health and social care professionals further complicated the feasibility of joint assessments. Although co-location was perceived by many as a positive development, it did not automatically facilitate changes to working routines. Even sitting arrangements within the same building often reflected professional boundaries, with nurses and therapists sitting in separate areas. In most cases, attempts at co-location of social care staff within health teams were hard to sustain, because of social services’ lack of capacity and financial resources. The interview excerpt below suggests that even where this was implemented, staff continued to work in silos, with different management lines and organisational priorities, which hindered genuine integration.

I think in terms of co-location, we are co-located but that does not always mean we are working collaboratively. I think being able to access our colleagues, being in the same room is easier, and it could speed things up. But I don’t think… I think there is still quite a definite rule for Social Care. I think the boundaries haven’t crossed. I think people still work in their own kind of… Their professional limits rather than… I don’t think there is any sort of crossover. (Nurse)

In some cases, where there was just one social worker co-located with health workers, they would feel isolated, because of different professional cultures.

At the beginning I asked my manager [to be co-located with health care professionals] so I would be able to work from their system and everything, and they set everything up, but I prefer to work from here because there are things… […] I would be very isolated and I need advice from my colleagues very often, so if I had to call every time and tried to, you know, to fathom out what they are doing, it would be much more difficult. So I prefer to be based here. (Social Worker)

Participants mentioned that relatively new roles in the community teams, such as care navigators, took years to be recognised by other professionals. At the time of fieldwork, they finally appeared to be embedded in the system, and both DNs and GPs increasingly relied on them as a bridge between different clinicians and the patient.

I think there is more demand now. I think over the years as our role has embedded itself, we are getting… People… rely on us is perhaps the wrong word but we are very much a part of the MDT and very much the go to people when you have got an issue. ‘Well, we don’t know what to do with the patient’, ‘Speak to the Care Navigators and they will come up with something.’ [Care Navigator]

On the ground the staff absolutely love working in an integrated way. We feel we’ve not achieved it, particularly with social care, but we love working with therapy and palliative care and care navigators. [DN]

These findings support existing literature that has recognised care navigators as an important mechanism of coordination of patients with multiple needs [33]. In this respect, the decision to reduce their numbers in some of the localities under study was perceived by many participants as another example of how far removed top-down change can be from the day-to-day reality of service delivery.

### Discharge Pathway

Discharge pathways were particularly complex in all three municipalities, with overlap between new teams and existing ones, and poor coordination between acute and community care. While there was much focus on Delayed Transfers of Care, with investment in consultant-led projects in all main hospitals across the three sites, the interviews highlighted concerns about patients being discharged too early or without the required medication, leading to hospital readmissions. Physiotherapists and OTs often mentioned that increasingly patients were being discharged when “medically fit” but still needing high levels of reconditioning and rehabilitation, which community teams or D2A services were not always able to deliver. This was also perceived to be a consequence of broken communication between community teams and ward staff, as the latter had limited understanding of community pathways and community provision.

So the challenging experience on that is the therapists on the ward, they tend to discharge the patient’s heavily relying on us in the community, so their duty of care from the ward is not really… what do you call this? They don’t seem to do much in terms of their duty of care from the ward, because for example they will discharge the patient straight to the community without thoroughly assessing the patient’s needs, because they are relying on the therapists from the community. […] So one great example is: they will discharge the patient without the appropriate equipment, i.e. a hoist. They identify the need that the patient will need a hoist, but they will send a patient home anyway, because [we] will pick up the patient and will do the assessment. And I guess that’s part of the discharge to assess model, but then again my argument is as a therapist from the hospital, you identify the need, why don’t you facilitate that need for equipment and then request a joint visit with the [community] therapist because ideally that should be how it works. (D2A Occupational therapist)

Several participants identified specific roles that should be strengthened in order to help bridge the gap between hospital and community services, such as *in-reach nurses*. These are nurses with a community background working in the hospital in a community capacity and employed by a community services trust. The introduction of this role in hospitals was an attempt to facilitate coordination between the hospital and the community and improve patient discharges. However, where this role existed, it often had limited capacity and recognition in the hospital. In-reach nurses often did not have enough resources to appropriately cover hospital wards and attend all relevant meetings and board rounds.

The In-Reach team can only cover few wards in this big hospital and sometimes what they are expecting from us can be very difficult to achieve. (In-reach nurse)

Furthermore, while their role was appreciated by ward nurses in particular, in practice they had limited visibility and influence at board rounds. They often lacked adequate workspace in the hospital and this affected their self-efficacy, as they perceived this to reflect a lack of recognition of their role from the host organisation.

Observations revealed ways in which frontline clinicians and other health and social care professionals try to create new spaces to improve coordination on their own initiative. In Locality A, Discharge Forums were held monthly to discuss complex discharge cases involving people in different roles and from different organisations. The meetings took place at the local hospital and involved staff from acute care, community services, GP practices, social care, and the voluntary sector. These meetings represented an opportunity to discuss live cases by looking at the whole pathway with a focus on patient journeys; to develop an understanding of the difficulties encountered by the different health and social care professionals involved; and to suggest ways of increasing mutual support and improve communication. However, participants complained that a lack of support from management within some of the organisations involved signalled that the meetings were not a priority, and this inevitably affected attendance from their staff [34].

## Discussion

By looking at the micro-mechanisms of communication and collaboration of frontline professionals and their working routines, this paper offers a novel perspective on integrated care. The evaluation unveiled strong commitment to joined-up care among both health and social care professionals. The ethos of coordinated work is highly valued, as highlighted by other literature [35,36,37]. However, in the current context, overstretched staff were often forced into task-orientated and silo working, as they responded to differing organisational priorities, which, although seemingly aligned across organisations at senior management level, continued to be different, and at times conflicting, at the point of delivery.

Across all three localities, changes to services were not adequately communicated and understood and the pace of change was often perceived to be too fast. In many cases, the role of both RR and D2A teams was unclear to both hospital staff working on complex discharges and community teams. Participants often perceived these new services as disruptive or duplicating the work of existing teams, while many felt that investing resources in, and increasing capacity of, existing services, particularly in the community, would have been a preferable strategy. The pace of change in the NHS has accelerated in the last two decades [38,39], yet participants in the study did not feel they had influence over the change process. There was a general perception that new service reconfigurations were often introduced to mimic other organisations or to access available funding, without enough understanding of the local context and needs. This affected staff’s morale and decreased their commitment to engage with change processes. Increasingly separate acute/community careers and limited opportunities for rotation appeared to further deepen the barrier between the hospital and community services. The introduction of the new integrated services in many instances seemed to further exacerbate confusion.

Frontline health and social care professionals are at a vantage point to help commissioning bodies such as local authorities and CCGs to understand potential unintended consequences of, or existing barriers to, change. Bottom-up change has long being integral to the rhetoric of organisational development and integrated care, yet change continues to be top-down, overlooking needs and capacity at the point of delivery [40]. Our findings suggest that working closely with staff on the ground is paramount to support the development of new services and to strengthen existing ones, gaining better understanding of routines and implications of any changes at the point of delivery. The Discharge Forums described above are only one example of strategies led by frontline staff to strengthen integration that are proving more effective than recent top-down service redesign. Investing in this type of bottom-up change could ensure that the range of activities needed to generate system-wide cultural transformation reflects the actual capacity and needs of the organisations and systems [34,40,41].

## Conclusion

The rhetoric on integrated care remains strong. This is not surprising – as people live longer, they are also more likely to develop complex co-morbidities and require care from a number of different health and social care services over a longer period of time. Increasing specialism and fragmentation of health and social care are often a hinder to the required holistic approach to health and social needs. However, evidence of the effectiveness of integrated care as currently implemented is weak and our findings offer a contribution to the international literature in trying to explain the persisting gap between ambitious visions and the reality of frontline service delivery, or the difference between care as imagined and care as done [14]. Integrated care cannot be a way of managing austerity and requires upfront investment in both health and social care, while the latter has suffered particularly deep cuts in the past decade. Whereas staff in the NHS and social services have demonstrated remarkable resilience, in a context of increasing demand and decreasing resources, it is clear from this study that this approach to ‘making do’ is affecting the quality and coordination of the care provided. Participants in our research felt that, without the required financial and human resources, as well as greater alignment of different organisations’ priorities, committing to genuine and sustainable integrated care was unattainable, and many described the work of their understaffed teams as “firefighting”.

The pace of change in public services in recent decades has failed to take account of effective capacity of the ground. Change within any complex and highly hierarchical organisation such as the health and social care systems takes a long time to embed and requires a strategy of incremental change within a consistent broader vision. The literature agrees on the effectiveness of a bottom-up approach where the purpose and benefits of the change are understood and embraced by, and co-produced with, frontline professionals [40].

Several limitations of this study should be acknowledged, including the focus on one relatively small geographical area and the timing of the evaluation. The smallness of the sample size for each locality may also have masked some local differences. Some of the intermediate care services evaluated were still at pilot stage and some teams were undergoing new reconfigurations, which raised some confusion when discussing specific roles and activities with participants. Some of these limitations were addressed by organising several workshops with different teams to co-interpret and refine the findings.

Further research is needed on working routines of health and social care professionals, on how initiatives that aim to foster integrated care interact with these routines, and how the latter adapt or resist to change. These contextual micro-mechanisms will play a crucial role in translating vision into practice.

## Additional File

The additional file for this article can be found as follows:

10.5334/ijic.5432.s1 Appendix. Maps of Admission Avoidance and Discharge Based on Findings.

## Appendix

Services under study and interviewees.

**Table:**

Locality	Rapid response	Discharge to assess	Community services teams	Other

**A** **36 interviews** **(including 1 group interview with two participants)**	3 nurses; 2 physiotherapists (who also worked on Discharge to Assess) 1 service lead	3 occupational therapists 2 physiotherapists 4 social workers (including 1 service lead) 5 members of the reablement team – social services (2 service leads; 3 care workers)	3 district nurses (including 1 service lead) 2 physiotherapists (including one service lead) 1 occupational therapists 1 care navigators 1 social worker 1 GP	3 hospital clinicians 1 Occupational therapist 2 in-reach nurses 1 hospital nurse 1 service lead hospital discharge team
**B** **22 interviews** **(including 1 group interviews with 3 participants)**	4 nurses (including 2 service lead) 1 physiotherapist 1 GP	1 physiotherapist 1 occupational therapist 1 social workers 1 Service lead enablement team (social services)	3 district nurses 2 physiotherapists 2 occupational therapists 2 care navigators 1 service lead	1 consultant 2 hospital nurses 1 hospital nurse (discharge team) 1 hospital social worker
**C** **23 interviews** **(including 3 group interviews, two including with 2 participants and one including 3 participants)**	3 nurses (including 1 service lead)	1 service lead 2 occupational therapists 1 physiotherapist 2 service lead reablement team (social services)	6 community nurses (including 2 service leads) 1 physiotherapist 1 occupational therapist 3 social workers (including 1 service lead)	3 hospital nurses 1 service lead hospital discharge team 2 support workers 2 social workers in the hospital
