# Supplementary material for: (Dis)Integrated Care? Lessons from East London
Source: Int J Integr Care. 2020 Oct 19;20(4):2. doi: 10.5334/ijic.5432 (PMC7597578; doi:10.5334/ijic.5432)

APPENDIX – MAPS OF ADMISSION AVOIDANCE AND DISCHARGE BASED ON FINDINGS

Figure 2. Admission Avoidance pathway in Tower Hamlets

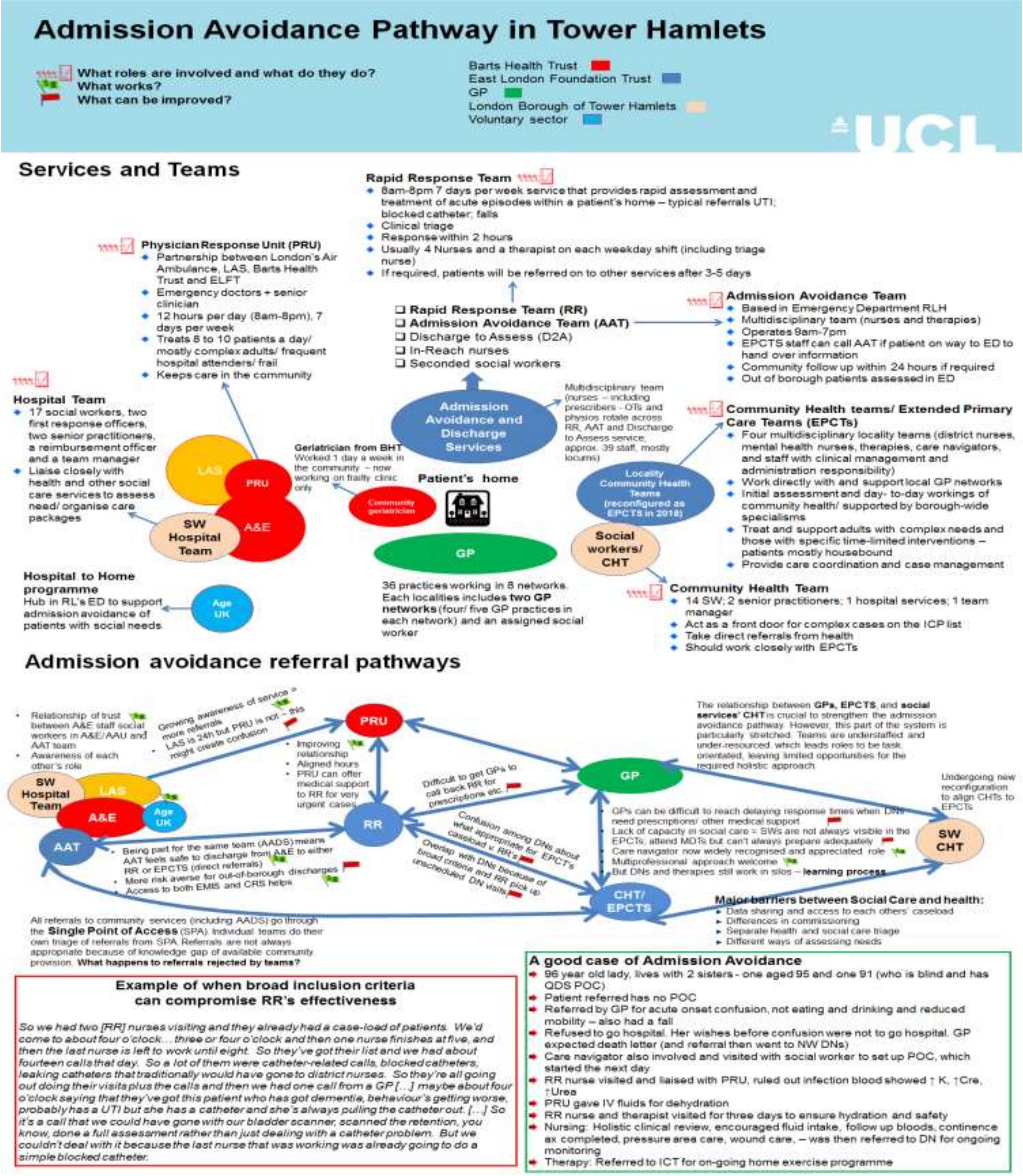

Figure 3. Admission Avoidance pathway in Newham

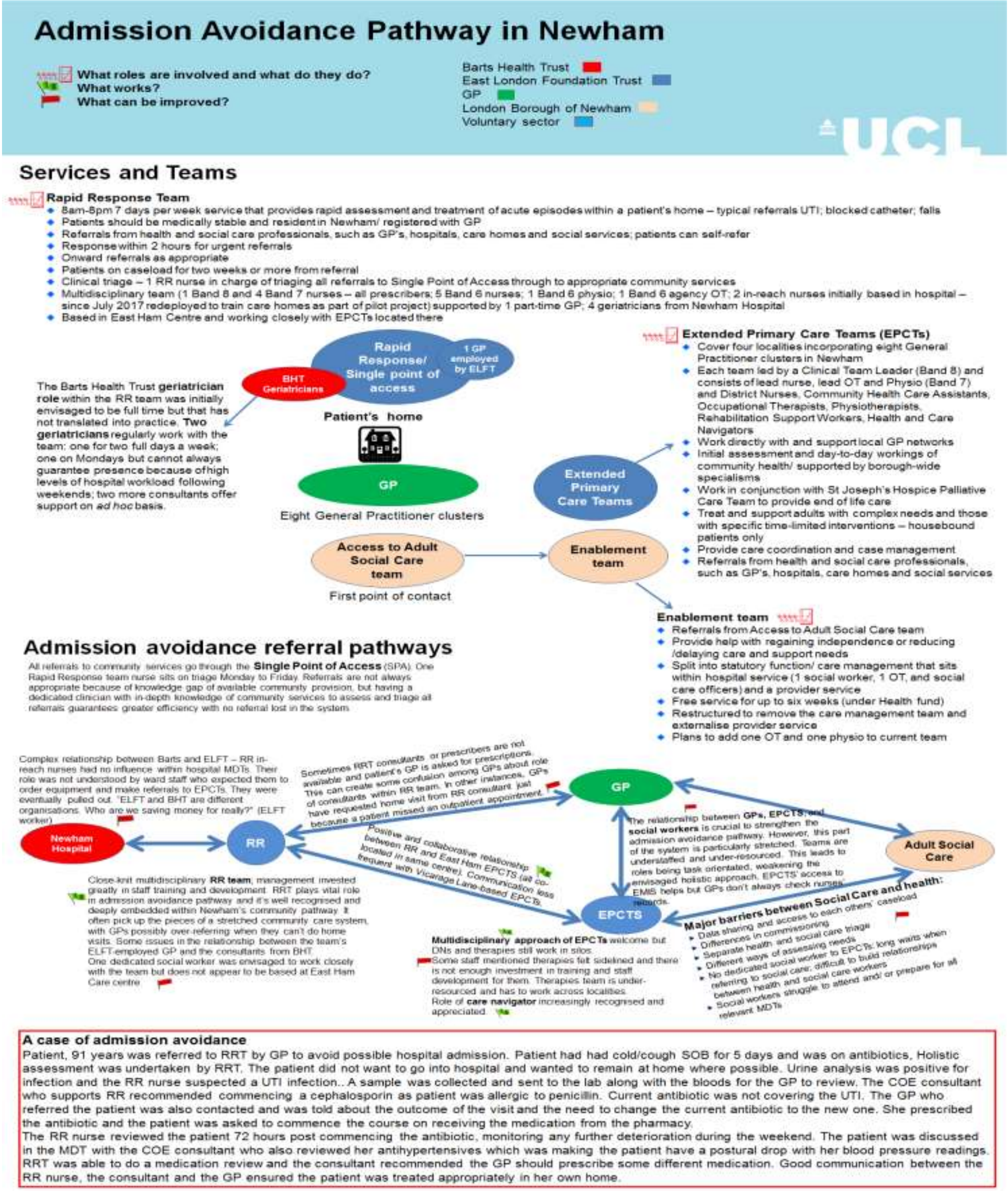

Figure 4. Admission Avoidance pathway in Waltham Forest

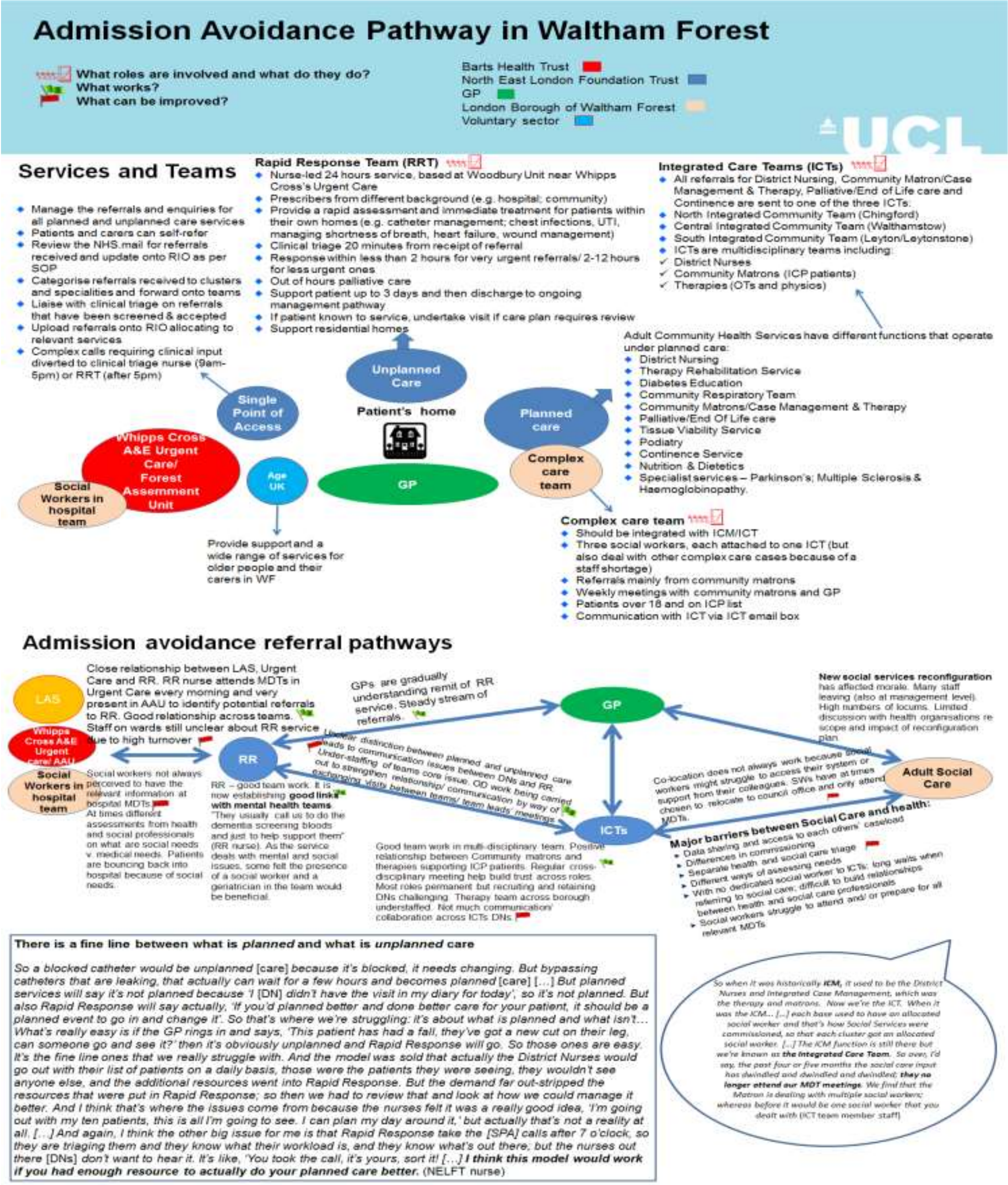

Figure 5. Discharge pathway in Tower Hamlets

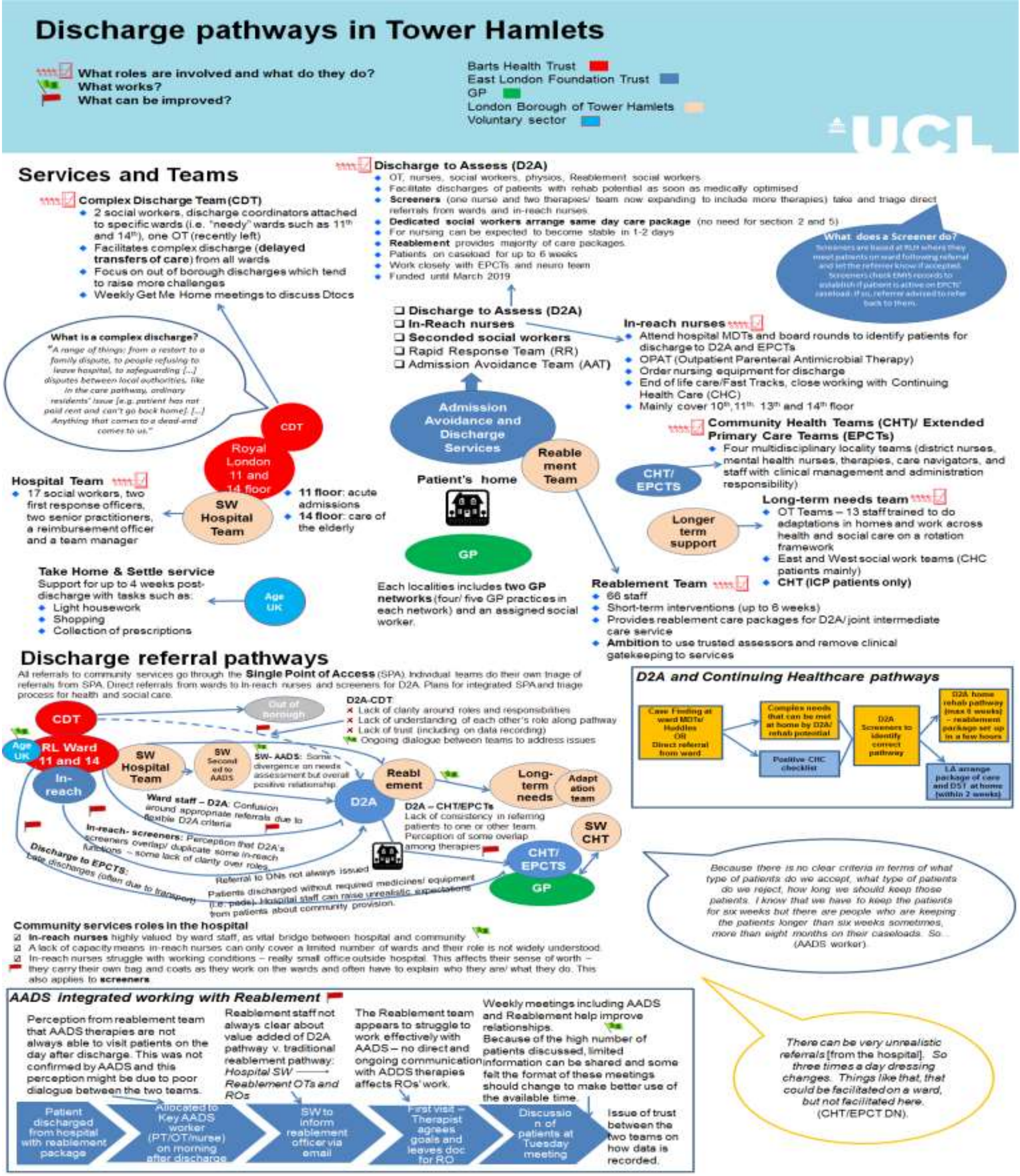

Figure 6. Discharge pathway in Newham

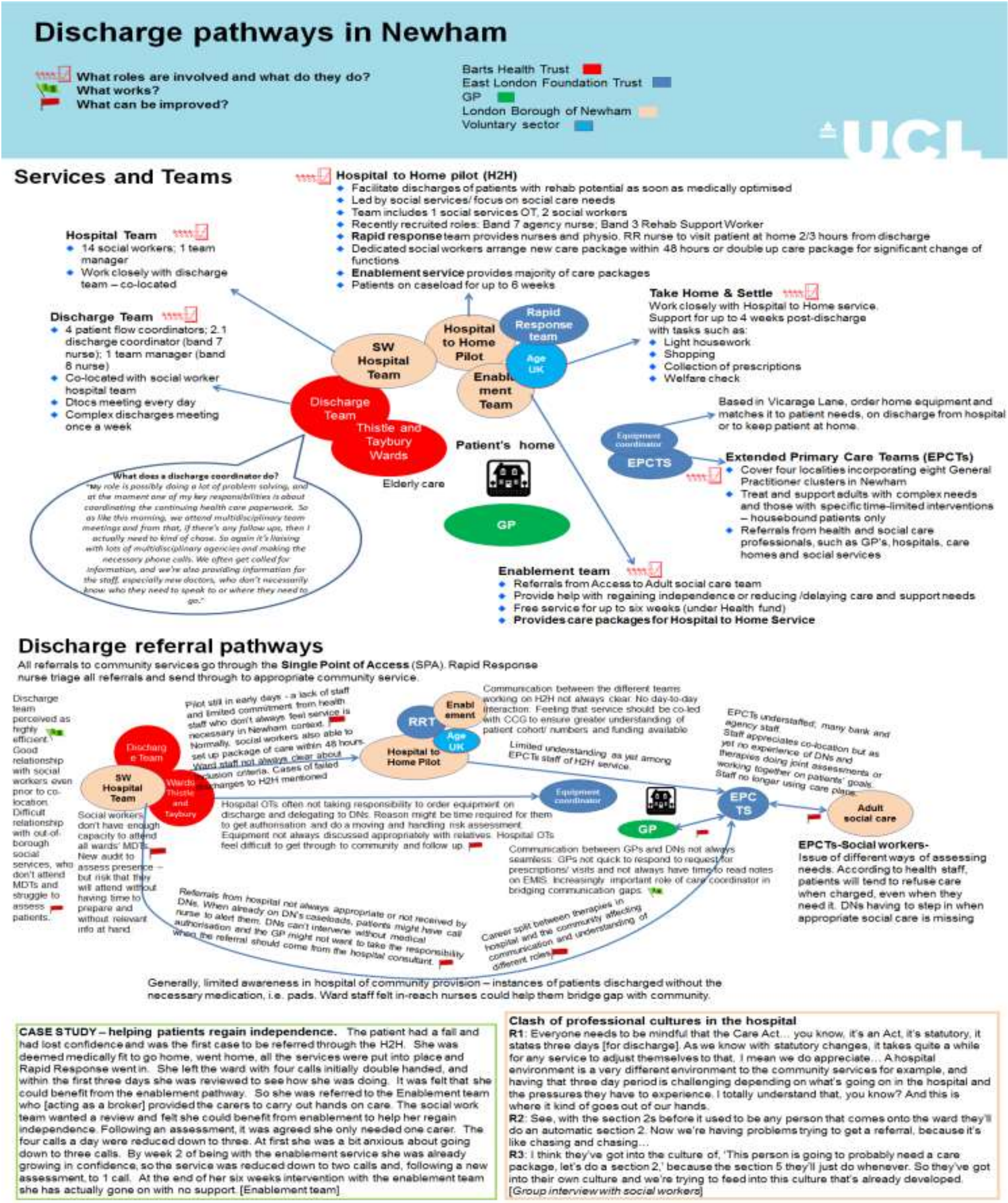

Figure 7. Discharge pathway in Waltham Forest

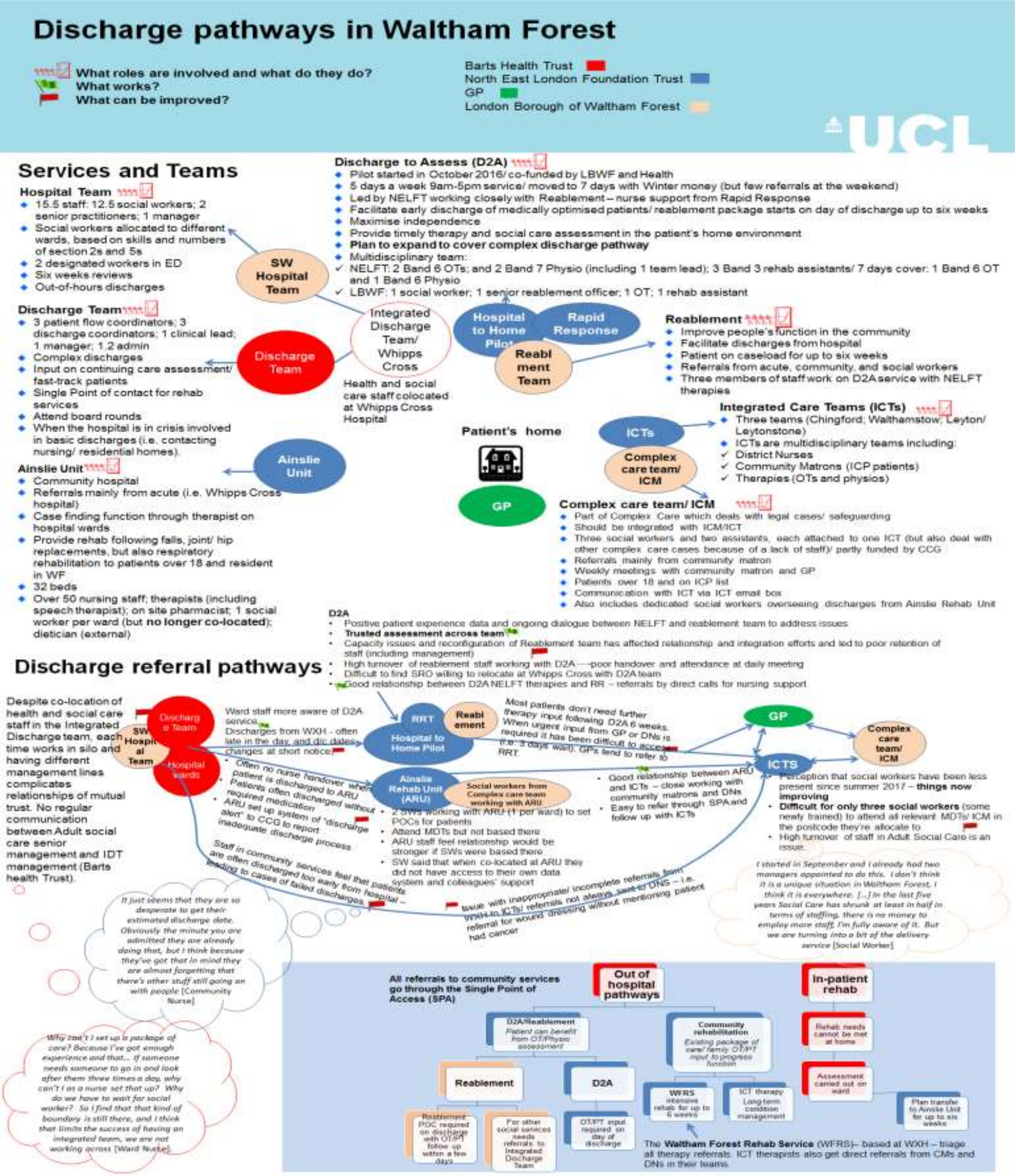

Supplement: Appendix. — Maps of Admission Avoidance and Discharge Based on Findings. [file ijic-20-4-5432-s1.pdf]
